# “I Have Been Born, Raised and Lived My Whole Life Here” – Perpetually on the Move While Remaining Still

**DOI:** 10.1007/s12124-021-09660-6

**Published:** 2021-11-16

**Authors:** Oliver Clifford Pedersen, Tania Zittoun

**Affiliations:** Institute of Psychology and Education, University of Neuchâtel, Neuchâtel, Switzerland

**Keywords:** Imagination, Mobility, Staying, Life course, Case study

## Abstract

This article explores the story of Einar, a Faroese man who always lived within a 500-meters radius on the island of Suðuroy, who never felt “stuck” or “immobile” in the literal sense of the word. Studies have shown that staying is a process, as much as mobility; yet while mobility studies mainly show that imagination is an incentive to move, we argue that imagination may also actively support staying. Combining sociocultural psychology with mobility studies, we propose to explore the entanglement of symbolic mobility (a form of imagination) and various forms of geographical (im)mobility. Based on ethnographic fieldwork and hours of conversation, we present the case study of Einar’s life on his island. We follow the sociogenetic development of the island, and the expansion and contraction of the imaginative horizon over time. On this background, we then retrace the life of Einar and show how, within this transforming context, his imagination developed thanks to resources he could use from the mobility of technologies, ideas, and other people. Interestingly, at different bifurcation points, his symbolic mobility almost led him to move away but, at another point, helped him to refuse geographical mobility. Hence, he was always symbolically mobile while staying. We finally propose directions for generalising from this case study, and implications for cultural psychology and for mobility and migration studies.

## Introduction

This article presents parts of Einar’s life-story. Einar is a Faroese man in his 90s who chose to live in the same village, Tvøroyri, on the small island of Suðuroy – one of the 18 islands that constitute the Faroe Islands, a small archipelago nation located in the North Atlantic Ocean – for his entire life. Oliver first met Einar in 2019 at a communal event on Suðuroy. A friend, who had brought him and a colleague there, pointed to an elderly man with bright white hair and large square glasses, sitting at the edge of the crowd and said, “you must speak to him”, explaining that he had never moved away from the island. She introduced Oliver to Einar, who is incredibly lucid, curious, speaks at least three languages fluently, and has an impressive knowledge of the Faroese history. Over the period of a year, Oliver met Einar several times and spent many hours talking about living on the island, travels, significant events, and, importantly, what made him stay at different points of his life. Although Einar sought to move away a couple of times, social and affective ties and a progressive differentiated imagination led him to stay in the village – a place marked by high incoming mobility on one hand, and emigration on the other. His story demonstrates that staying is a continuous process (Hjälm, 2014), and we will explore how his unique position at the juncture of mobilities and language skills enabled him to be symbolically on the move. Here we propose to consider relative geographical immobility as facilitated by imagination, and as it emerges from, and is entangled with, other forms of human as well as non-human (im)mobilities.

Studies on migration and mobility have shown the importance of potential movement – people’s imagination of what moving might bring about. Concepts such as *aspirations* (e.g., Carling & Collins, 2018; Carling & Schewel, 2018; De Haas, 2014), *imaginaries* (e.g., Salazar, 2011, 2014), or *hope* (e.g., Hage, 2009; Kleist & Jansen, 2016; Mar, 2005), in different ways capture the fact that what mobility promises may set people in motion. Such concepts centre predominantly on the outcomes of imagination (Cangià & Zittoun, 2020; Salazar, 2020). However, as sociocultural psychologists, we seek to re-orient the focus from the products of imagination to the process of imagination as it develop across times and spaces (Zittoun & Gillespie, 2016), at the intersection between life trajectories and social transformations. In other words, we are interested in the formation and development of imagination in and through mobility, which can manifest as aspirations, hopes or related terms. We aim to combine insights from a sociocultural psychological approach to imagination, with a mobility lens (Sheller & Urry, 2006) in order to explore how staying – as much as actual migration and mobility – entails imagination. Considering the role of imagination in the study of geographical mobility shows that imagination can both hinder or promote mobility, and that in turn, mobility can transform imagination (Zittoun, 2020). It also invites us to retrace the interplay between mobilities and imagination: People can remain in one village, yet be symbolically very mobile through imagination, or they can experience repeated mobility, but have their imagination fixed (Zittoun, 2020). Here we go one step further and suggest that imagination does not just develop as people move, but also as their lives becomes entangled with shifting mobilities of people, goods and ideas in a given place (Cresswell, 2006). We therefore look at diverse mobilities and their entanglements (Heil et al., 2017; Kleist, 2020). Altogether, through an exploration of imagination and entangled mobilities, we will explore what role imagination plays in Einar’s repeated decisions to remain on Suðuroy.

We first introduce the emerging literature on staying in mobility studies and suggest that it has paid little attention to imagination so far. We proceed to define a sociocultural psychological interpretation of imagination, which characterises it as a dynamic process, simultaneously social and individual. However, since research of imagination sometimes appears dissociated from mobility studies and the context in which it unfolds, we introduce the notion of ‘imaginative horizons’ (Crapanzano, 2004) to re-situate imagination in the economic, socio-cultural, and material conditions of its emergence. We then provide a description of Suðuroy, its history, and its evolving imaginative horizon, leading up to the point where Einar’s story begins. Following his story, we finally demonstrate that human and non-human mobilities do not only produce imaginations of geographical mobility, they can also be used to imaginarily explore the world while remaining put.

## Staying as a Dynamic and Active Process

In a world assumed to be perpetually on the move (Adey, 2017; Cresswell, 2006), those who stay put are often perceived as left behind (Jónsson, 2011) or as having failed to leave (Looker & Naylor, 2009). However, in line with increasing attention to processes of staying (e.g., Schewel, 2019; Stockdale et al., 2018; Ye, 2018) and immobilisation (Glick Schiller & Salazar, 2013), we, too, aim to demonstrate that not moving is much more than a simple absence of geographical mobility. According to Hjälm, staying “is not a decision that is made once and never renegotiated, and it does not occur in isolation but is connected to other life projects and people” (2014, p. 579).

We adopt a mobility lens (Sheller & Urry, 2006; Urry, 2007), which enables us to consider mobility beyond the national container (Anderson, 2019; Dahinden, 2016; Malkki, 1992; Wimmer & Glick Schiller, 2002). Therefore, we want to emphasize the messiness and interdependencies of the many forms of movement and stasis that become meaningful for people at different points during their lives (Cresswell, 2006; Halfacree & Boyle, 1993; Kalir, 2013; Khosravi, 2010). In other words, we aim to transcend notions of “distinct ‘places’ and ‘people’” (Hannam et al., 2006, p. 13; see also Ferguson & Gupta, 1992). As Adey proposes, “mobility and immobility are profoundly relational and experiential” (2006, p. 83): it is through speed differentials (and hence relation) that mobility and immobility attain meaning, which can be called “relative immobility” (Adey, 2006, p. 84). Cresswell makes a similar point in suggesting that “some mobilities are dependent on the immobilities of others” (Cresswell, 2001, p. 21), viewing stillness as an integral part of moving (Cresswell, 2012). Here we are not simply referring to the speeds at which a person moves across time and space, but also to the person’s relation to the mobility of other people, goods and ideas (Cresswell, 2010; Söderström et al., 2013). Mobilities and immobilities are entangled and each constellation or regime engenders or restricts processes of moving or staying.

The process of staying happens at this entanglement between mobility and immobility, and stayers are not “passive observers of their own fates” (Stockdale & Haartsen, 2018, p. 2). Frameworks such as those centring on aspirations also seek to capture the agency of people and to bridge the micro and macro level of analysis (Carling & Schewel, 2018; De Haas, 2014). However, these models tend to retain a migration-centric perspective, they emphasize outcomes over processes, and they do not trace the dynamic development of imagination that occurs between and across level of analysis. Through our sociocultural psychological approach to imagination combined with a mobility lens, we hope to widen the scope of inquiry to include all kinds of mobilities, we wish to illustrate the process through which imagination of movement and stasis arise in specific (im)mobility constellations and life-trajectories, and to provide a systematic programme to unravel sociogenetic and ontogenetic convergences and divergences. This is not to say that people cannot be or feel stuck in immobility (Coulter et al., 2016), detention centres (Griffiths, 2014), movement (Wyss, 2019) or are always in a position to realise migration aspirations (Carling, 2002). Rather, we view staying as heterogeneous category encompassing “passive and active, unintended and intended, and the many in-betweens” (Ye, 2018, p. 8), entangled with different mobilities and which changes over time.

We also recognise that other factors, such as social and economic ties (Fischer & Malmberg, 2001; Mulder & Malmberg, 2014), attachment to local community and place (Barcus & Brunn, 2009), histories (Rérat, 2014), social and affective ties (Cole & Groes, 2017; Schapendonk, 2020), regimes of mobility (Glick Schiller & Salazar, 2013), and more shape and are shaped by mobility and immobility processes. Nonetheless, research on staying and stayers have yet to explore what role imagination plays in the process beyond the sociocultural imaginaries of what staying means (Mata-Codesal, 2015, 2018). Research has shown that imagination can drive mobility and migration (e.g., Baas, 2010; Benson, 2012; Pine, 2014; Salazar, 2014; Vigh, 2009), be triggered or blocked in mobility and migration (Cangià, 2020; Womersley, 2020), and not always correspond to geographical mobility (Salazar, 2010; Zittoun, 2020). As Salazar (2011) write, the interesting question is not how much imagination corresponds to actual movements; it is rather how contexts conditions imaginings. We will explore some of the ways it restricts or engender staying and vice versa.

### Two Types of Mobilities

The concept of mobility designates a broad range of phenomena and types of movement far beyond humans merely moving in space. We do not aim to provide an exhaustive list but seek to distinguish geographical from symbolic mobility. *Geographical mobility* denotes any form of movements – of people, technologies, ideas and goods, and so on – that cross time and space. These happen at different speeds, follow different routes, and are experienced differently (Cresswell, 2010). *Symbolic mobility*, on the other hand, captures the movement experienced through imagination; one can imagine traveling without actual geographical movement (Zittoun, 2020). People can imagine faraway places and living elsewhere through talking to people, listening to podcasts, seeing documentaries, reading travel blogs, and so on. In other words, through the process of imagining people can move symbolically. People can also be very geographically mobile yet symbolically immobile and be immobilized but explore distant places and futures symbolically – as might be the case during a global pandemic. People’s capacities to be symbolically mobile depend on access to symbolic and economic resources as well as existing power structures (Marková, 2017). Symbolic mobility is connected to other forms of mobilities; very often, imagination is a central part of and develops with geographical movement. It enables us to understand why some people become mobile while others do not, how people deal with being forcefully stuck, as well as how they can satisfy a curiosity for the world while staying. From this perspective, particular entanglements might restrict one type of mobility while engendering another; yet these configurations are dynamic and evolve over time. Through this paper, we will also mention *social mobility* to designate the process by which people are moving upward (or possibly downward) the socio-economic matrix, however we put less emphasis on this more widely studied phenomena (Faist, 2013).

To contextualise the entanglement of different mobilities, and their relation to imagination in a specific setting, we turn to the concept of imaginative horizon (Crapanzano, 2004). It is a dynamic and open concept that relates to what is possible (Glăveanu, 2020a; Zittoun et al., 2020).

## Imagination and its Horizon

From a sociocultural psychological perspective, imagination is the process by which people temporarily disengage from the here-and-now of their actual physical and social location, to explore past, future or alternative ones (Zittoun & Gillespie, 2016). Imagination is what allows people to engage with what does not exist (anymore or yet), to embark on moving or to staying put, to explore faraway place while remaining still, or to feel anchored in a specific place while moving fast (Easthope, 2009), as well as to explore, play with, and expand what is possible (Glăveanu, 2020b). However, imagination is not an unbound, purely psychological process; it is always constrained, steered and facilitated by the sociocultural, economic, institutional and material conditions in which it takes place (Zittoun & Gillespie, 2016).

The historical and sociocultural settings in which imagination takes place create a horizon of what can and cannot be imagined (Zittoun & Gillespie, 2016) – figuratively and normatively. It also informs whether or not an imagination can be actualised or not. The notion of “imaginative horizon” has been proposed by Crapanzano (2004) to describe the dialectic between reality and its “hinterland”, the beyond as the site from which possibility arise. The concept of imaginative horizon offers a useful analytic tool for capturing what can (and ought to) be imagined in a given setting, and whether an imagination is actualisable. Of course, such horizons are never static and Ingold captures this ephemeral and perspectival nature in suggesting that they can never “be reached or crossed since, like the rainbow's end, they move as you do” (2008, p. 2).

Imaginative horizons have various properties. First, they are constituted by material and symbolic factors, which affords certain possibilities for imagining. Here, we will focus on the economic system, infrastructure, mobilities and sociocultural conditions as part of what shapes the imaginative horizon. Second, they develop through time. To account for their dynamics, we propose to consider that imaginative horizon may be more or less centralized, or more or less expansive. Centralisation represents the gravitational pull in a given horizon; it can be due to salient features of the socio-material landscape or to strong normative forces that steers people’s sense of possibility. Centralisation typically occurs when some institutions or authorities control access to resources for imagining, or control people’s modes of imagining (Hawlina, 2019; Marková, 2017; Zittoun & Gillespie, 2018). Expansion, in contrast, denotes the non-linear or non-teleological transformation of the imaginative horizon.

What then is the relation between people’s imagination and imaginative horizons? As sociocultural psychologists, we assume that people located in a given sociocultural and historical setting are actively appropriating and internalising available cultural elements; collective meaning are transformed in personal sense-making, depending on people’s unique trajectories (Bruner, 1990; Rosa & Valsiner, 2018; Valsiner, 2007; Zittoun et al., 2013). Similarly, imaginative horizons furnish people with symbolic material, actual opportunities and afford certain imaginations. Yet what people will draw upon, and imagine within these boundaries, or at their very edges, depends on their unique perspectives, themselves changing along their life trajectories.

## Situating People’s Life Imagining within the Imaginative Horizon

To study the evolution of a given imaginative horizon, we need to study the history of a place and identify elements that participate in its making, including its expansion and centralisation over time. Only on this background can we understand Einar’s story and capture the evolution his imagination as it is afforded, supported or constrained by the imaginative horizon. These two evolutions are two analytical entries into developmental or historical dynamics.

At a first level, we can reconstruct sociogenetic dynamics – the evolution of the social field. This requires a reconstruction of histories, socio-material transformations, and mobilities. To reconstruct imaginative horizons, we proceed genealogically (Foucault, 1978; Gillespie, 2006) with the elements that we believe participate to the making of that imaginative horizon. Within the frame of the paper, this involves documenting the material circumstances and its symbolic counterparts. Places are naturally connected to the rest of the world and so we account for circulations on and through Suðuroy: Traveling and communication, mobility of goods and technologies, in and out mobility of people, and with all of this, circulation of languages, discourses, ideas and social representations (Appadurai, 1996; Glăveanu, 2020a; Massey, 2005; Urry, 2007). All these elements, we propose, participate to the construction and transformation of the imaginative horizon. They also create opportunities to be mobile, socially, geographically, or symbolically, or impose, control, or restrict these in certain conditions. To limit our enquiry, we focus on aspects of these sociogenetic events that seem relevant for Einar’s story.

At the second level, we document the life trajectory of a single person – their ontogenesis (Elder & Giele, 2009; Hviid & Villadsen, 2015; Valsiner, 1998; Zittoun et al., 2013). For this, we reconstruct the chronology of Einar’s life, which we situate within a larger history (Gillespie & Zittoun, 2015; Kohler-Riessman, 2000). Then, based on Einar’s story, we can identify points of bifurcation (Sato & Valsiner, 2010; Sato & Yasuda, 2013): Particular moments where Einar had the possibility to move or stay, and the individual and social forces involved. At each of these, we examine the real and possible mobilities: Is it about geographical or symbolic mobility? Is it about Einar moving, or movement of other people or goods, or is it only happening in imagination? In addition, we look at all the elements, which we believe can feed into people’s imagination: Access to social representations and practices, as well as to more personal symbolic resources, such as meeting with people, and experiences of mobility – one’s own or that of others. Indeed, the more people have experiences and access to elements likely to become resources, both personal and socially shared, the more their imagination can be deployed and precise (Vygotsky, 2004; Zittoun & Gillespie, 2016).

In this paper, we explore the interplay between the transformation of the imaginative horizon on Suðuroy in and through mobility, and Einar’s life and im/mobility trajectory (Schapendonk et al., 2018). Our arguments are, first, that although people’s mobilities have to be understood on the hinterland of a socially, historically and spatially located imaginative horizon, they have space to define their imagination and mobile trajectories. And second, we argue that people’s mobilities may be more complex that they seem: An apparent relative geographical immobility may reveal complex and diversified patterns of social and symbolic mobility.

Methodologically, we thus work at a double level in attempt to bridge ontogenesis and sociogenesis. On one hand, we rely on more than 3 months of ethnographic fieldwork conducted over the period of a year, more than thirty qualitative interviews and extensive archival research (of histories, demographics, ethnographies, media, and so on) in order to build a dialogical case study (Marková et al., 2020) and, ultimately, warrant a genealogy of the imaginative horizon. On the other hand, to capture Einar’s story, we rely on Oliver’s relationship and hours of conversations with Einar. Oliver recorded two biographical interviews with Einar, each about 3 hours long, in which Einar was asked to tell his life trajectory and experiences in different periods. Einar’s narration is often gendered as it centres on men – likely reflecting his experiences growing up in a men’s world – and thus we have decided to focus on that side of the imaginative horizon. Oliver furthermore visited Einar a couple times more without recording but noting down the key points. At almost every visit, the conversation was interrupted by the comings and goings of Smyríl or of foreign ships. Einar lives on the mountainside with a couch placed in front of a big window with a panorama overlooking the fjord and ferry terminal on the other side of the fjord. Situations like this, or the time where Oliver and Einar sat at dusk without any lights on, while the latter explained how generations before him always referred to this time of day as the best time to talk, not only forged a relationship but also revealed something about the island and its rhythm. Oliver continuously informed Einar about the intended use of the interviews and the notes written– Einar consistently consented. However, we recognise that writing a story about one person from a small village on a small island naturally poses concerns about first and second order anonymity. People from the island would easily be able identify him even when covered by the thin veneer of pseudonyms. Oliver therefore shared the final draft with Einar before submitting it to get his comments and re-affirmation that he still was fine with it being published after having seen the end-product. In the version Einar received, we had anonymised his name. After reading the article and discussing it with his daughter, Einar expressed a desire to have his name. He wanted people to be able to read his story. Oliver called again a few weeks later to discuss the possibilities anonymising it and talk about the loss of control upon publication – again Einar thought it would be best to keep his name. We have therefore decided to follow his wish. Further, on Einar’s request, we have also translated the article into to Danish so he can disseminate it to his family and friends. Some of the more sensitive subjects are deliberately left out, which might give the story a positive twist, but also does away with some of the most pressing concerns about not anonymising. Oliver planned to return and talk more with Einar, however due to the COVID-19 situation, it became (and when writing this, still is) untenable.

## A Brief History of the Faroe Islands

Instead of giving a complete roadmap of Faroese history, which others have done (e.g. Sølvará, 2020; West, 1972; Wylie, 1987), this section presents a brief genealogy of the circumstances surrounding the parts of Einar’s story we will zoom in. We first briefly discuss the transition from feudal to fishing-based society because it has played an immense role in in shaping emerging mobilities, infrastructures, socio-economic life, and imaginations.

Until the early-nineteenth century, Faroese economic and social life remained almost cashless and centred on agriculture. Fishing, whaling and fowling served as additional means of subsistence. Steep mountains, unpredictable weather and a treacherous sea restricted mobility between villages and islands, and emigration remained miniscule. This is said to have created somewhat self-contained micro-cosmoses – worlds within worlds – living according to seasonal rhythms and at the whim of the weather. Legislation further restricted the mobility of the peasant class (Brandt, 1983; West, 1972), which constituted a large part of the population, and limited their ability to start families (Wylie, 1987). The advent of fishing contributed to breaking this fatalistic bond and introducing the promises of mobility.

Joensen (1987) distinguishes two phases of the fishing-related industrialisation. First, near-shore fishing from around 1840 and, second, smack fishing from 1872. The first phase was, in parts, facilitated by the Royal Trade Monopoly’s decision – all trade to and from the Faroe Islands was controlled by this Danish institution – to start buying and exporting fish from local anglers due to the declining price of wool products worldwide (Joensen, 1987). It was further enabled by the adoption of fishing technologies developed by the Shetlanders. Here we observe how the transnational mobility of technologies and capital creates new routes for people to be mobile – that is, an entanglement of human and non-human mobilities. We characterise this period as one in which the imaginative horizon expanded and increasingly turned the imagination of formerly landless peasants towards the sea, to new forms of mobilities and possibilities; yet old rules mandating peasants to be available for operating the farmer’s boat kept them in place. Then, in 1856, the Royal Trade Monopoly was dismantled and sold off to would-be merchants adding pressure to already fragile feudal structures. The boat-tie system was abolished in 1865 (Brandt, 1983), legally allowing people to pursue professions on the sea and become mobile. Increasing internal mobility between islands seems to have created a heightened social awareness of what exists beyond the village, and created an urbanisation towards those villages with prominent fishing. The second phase began as British and Scottish anglers were modernising their fishing fleet, which enabled the Faroese to buy cheap smacks with the first acquisition taking placing in 1872. Altogether, this opened for new possibilities, in particular for a new working class who could now strive for social mobility as well. Workers had now gained the right to be mobile, which allowed them to take work on other islands or boats and buy or lease small plots of land in order to establish families. Fishing rose to become the sole most important industry in the Faroe Islands and re-configured what forms of mobility was imaginable and actualisable, as well as dictating the rhythm of social life.

Suðuroy, where Einar’s story takes place, was, in many regards, relatively isolated and peripheral in the Faroese socio-political cosmos before the Royal Trade Monopoly established a branch on the island in 1836. Sheer geographical distance, method of transportation (rowing boats), and the Faroese weather made the trip to Tórshavn (the capital city) long and potentially perilous. During the stormy winter months, the journey was simply not possible and, still today, I have experienced being ‘stuck’ on the island or not able to return due to the forces of nature. In a second phase of the fishing adventure, two villages on Suðuroy, Tvøroyri and Vágur, rose to become powerhouses in the Faroese economy (Holm & Mortensen, 2002). By the turn of the twentieth century, about 30% of the Faroese fishing fleet was based in Tvøroyri alone (Guttesen, 1996). This socio-economic development clearly shows in the population growth: between 1880 and 1916, Tvøroyri went from 468 inhabitants to 1522, and Vágur from 335 to 912. The total population increased from a little over 11.000 to over 18.000 in the same period (Wylie, 1987). Suðuroy became a site of entangled mobilities. Non-human mobilities, such as new incoming technologies (e.g., the first power plant in the Faroe Islands) and bettering infrastructure (e.g. first regular ferry connection), or the circulation of ideas of unionization, were engendered by and engendered new forms of human mobilities. These mobilities included, among others, anglers shipping out to fish around Iceland and Greenland, incoming labour forces from the other islands to help process the catch, and people leaving to educate themselves in Denmark (primarily children of the merchant class). Then came the Wall Street crises in 1929, which, combined with declining prices of fish throughout the mid-1930s, resulting from trade restrictions and wars on the European continent (Numminen, 2010), slowed down the pace. The Second World War created an unlikely opportunity for the Faroese to supply war-ridden Britain with fresh fish (Numminen, 2010).

In the 1930s – formative years of Einar’s childhood – the imaginative horizon for men on the island was to a certain extend still centred around fishing, which is reflected in the proportion of people directly or indirectly employed in the primary sector (Guttesen, 1996). Einar confirms that becoming an angler was a naturalised pathway. It was normatively expected of men to ship out after their confirmation or once school finished, while woman processed fish, raised children, and handled the household (Wylie, 1987). However, besides the men spending months on the Ocean, many still stayed relatively put. Outward mobility remained an event and still not everyone had access to it. Meanwhile, fishing jump-started an economic modernisation and diversification, giving rise to the emergence of new industries and professions (Wylie, 1987), producing a clash between two ways of life as Faroese writer Heini Brù eloquently captures:

“Over there, you can see study old men clad from head to foot in their thick homespun, their heavy whaling knives at their belts. These are the men who grew up at the oar, and trod out the mountain path. For them, all journeys were long and risky ones. They are all keyed up to meet any problem, and they take life very seriously […] And over there, you see the young fellows dressed in their seaters and overalls, with their cloth caps on their heads […] These are the men who built the roads and the landing stages, who learned to deck in their fishing boats and install motors in them. They measure time and distance differently from the older folk. Journeys are shorter for them, and time is not such a serious matter. These men are lighter-footed, lighter-hearted, and move lively-spirited than the older folk.” (2011, pp. 7–8)

While Brù’s description is a simplification, it alludes to something anthropologists have observed as well (e.g., Gaffin, 1996), and it metaphorically echoes Einar descriptions of life back then. For generations before and around Einar, for whom additional means of subsistence remained important for survival, versatility appears to be a valued trait because it brought food on the table and it meant being able to cope with the unpredictable. For instance, Einar describes the difference between a good and a bad winter in terms of having whale meat, and he adheres to a hard-working ethic whenever talking about work. As a consequence, foremen, clerks, and post people – to name a few – were sometimes portrayed as “doing nothing” and ridiculed (Gaffin, 1996). But, at the same time, the growing industrialisation also gave rise to jobs in the secondary and tertiary sector (Guttesen, 1996).

With increasing encounters with the world beyond the relatively self-contained villages, new professions as well as the gradual improvement of infrastructural technologies opened a new field of possibility, which we interpret as making imagining different lives more plausible (Appadurai, 1996). Things which were previously impossible to even imagine – like owning a house, getting a payed salary or finding specialised work – slowly entered into people’s imagination. As Einar remarks, and which statistics seems to corroborate, emigration was still not possible for most people. This expansion of the imaginative horizon implies that the future progressively seemed less bounded by fate, although material and economic circumstances still exhorted some constrains.

## The Curious Story of Einar

I have been born, raised and lived my whole life here in the village and I also want to say – I want to be buried here. It has been my intention since I was quite young. And why, I don’t know. I have no explanation.

Einar grew up during the period described above. The first years of his childhood, Einar lived in small house together with 12–14 people, but his family eventually moved to a house where he shared a bedroom with his two brothers until each of them were married away from the bedroom, as Einar puts it. While he recognises that his family had limited means when growing up, he did not experience this as a problem partly because the three brothers would frequently visit their aunt and uncle who owned a small farm, where they could get plenty fresh milk.

In what follows, we attempt to understand how Einar decided to stay in the village, which, at times, was characterised by increasing emigration. To this end, we present his life trajectory as chronologically as possible, based on a reconstructed timeline, emphasising two bifurcation points. These do not represent the entirety of Einar’s story nor are they given equal weight; rather, each enable us to highlight his staying and his engagement with various forms of human and non-human mobilities; they also represent steps in the development of his imagination.

Einar repeatedly told Oliver that he was born the year during which Lindbergh made his first solo trip across the Atlantic Ocean by plane. He also matched his brothers’ birth years with local and global events. For instance, Einar said that his oldest brother was born the same year as the local harbour was built. At first glance, we did not think much about this association, however, with time we came to hypothesize what it reveals about Einar’s mobility and immobility. While Einar’s oldest brother is symbolically linked to the centre of the village, Einar is symbolically mobile and crossing vast distances. This foreshadows Einar’s story as presented below: He always lived within a 500-meters perimeter, and even when he did consider moving away during his life, returning was always part of his imagination. Moreover, although he never emigrated, Einar was never immobile in the literal sense of the word, travelling between villages, islands, and later, also abroad. Yet, as we see, Einar was always also very symbolically mobile.

### Growing in Times of High Mobility and Expanding Possibilities

Einar grew up in the 1930s. His world centred on three places in the village; the harbour, the school and the club – a local gathering place for men. He enjoyed school and describes himself as a decent student, but adds “never more than that”. Despite growing up during turbulent years, Einar’s imagination of his childhood is filled with a sense of wonder. He describes the joy of roaming freely in the mountains and sailing unsupervised in the fjord. Einar spent a great deal of time describing how he, and the other boys (girls were not supposed to be at the docks, he adds), were captivated by the foreign ships arriving.

[…] Getting on board the foreign ships that arrived – we boys were very excited about – to meet the others on board and talk to the ship’s chef and we always got some candy to eat […] It was our gravitational point.

Suðuroy position as one of the fishing centres meant a lot of oceanic traffic, and Einar would always try to get on board the ships to meet the foreign anglers. Experiences like this might have functioned as windows to the world beyond the island’s natural barriers, as radio was still unreliable and newspapers scarce. This constant inflow of people (anglers) and goods (candy, equipment, etc.) seem to have provided with resources for imagining alternatives to the present – to imagine faraway places – with tangible and, sometimes, material implications. For instance, as he explains, it also brought in a lot of ideas and technologies, which further expanded the possibilities on Suðuroy as well (Glăveanu, 2020a). Take the construction of the first hydropower plant:

Those merchants – we just call them merchants, they were also owners of ships – they realised, together with foreign people, that this waterfall [on the southern side of Suðuroy] could be used to develop a powerplant.

At that time, fishing remained a centralising element in the imaginative horizon and in Faroese socio-economic life. As Einar’s experiences and historical accounts demonstrate, it was a force generating most internal, inward as well as outward, and transnational mobility. Except for the anglers who roamed the waters near Iceland and Greenland, or the merchants selling their catch to Mediterranean countries, geographical mobility was limited in those years. Of course, people did move between villages and occasionally between islands, although as Einar remarks, it was mostly in relation to specific events, such as a grind – the practice of killing pilot whales – or to attend a dance:

Not so much [asked if they travelled between the villages]. You did it if it was necessary. And, once in a while, you did it to visit family or if there was a special dance in a village.

In addition, visiting foreign countries or emigrating to Denmark were primarily within the capabilities of the upper class of Faroese society:

No, we did not think about that [visiting foreign countries], but we knew that those people, who had money around here, they sent their children […] to study in Denmark.

By contrast, Einar notes that there was a lot of Faroese arriving from the other islands to help process the fish or to work on ships because there was a labour shortage, and foreign anglers stopped on the way to or from fishing grounds further north. When available, Einar’s father encouraged him and his brothers to listen to the radio but the signal was unreliable and best at night during winter months.

Hence, during Einar’s childhood, the rhythm of everyday life, mobility and (real and imagined) possibilities were, to a large extend, centralised around fishing and its seasons. The capacity to realise an imagined mobility was, for many, constrained by infrastructure and the economic situation. At the same time, we propose that this myriad of entangled mobilities along with societal changes expanded the imaginative horizon, opened new pathways and provided people, like Einar, with resources for imagining. The parochial and fatalistic horizon that had arguably set the bounds of villagers’ imagination, in the sense of “not thinking about” emigrating because it appeared in the realm of impossibility, slowly began to expand outwards as encounters with the world increased and the socio-economic situation improved. This broadly fits the proposition made by de Haas and others (Haas & Fransen, 2018), for whom societal transformation and economic prosperity is said to increase people’s aspirations to migrate, yet, as we shall explore, also engenders staying.

In this context, we might expect Einar to imagine being mobile, but speaking about his childhood or upbringings, he reports no personal need to move away. Paradoxically, the expansion of the imaginative horizon and increasing capabilities to be symbolically mobile seem to have facilitated his staying. Living at the one of the epicentres of the Faroese fishing revolution ensured a continuous influx of people, technologies and goods:

We did not travel that much but it increased a little as time went past. We didn’t really need it [to be geographically mobile]. We had most things. Suðuroy was well covered.

Being a site of high mobility of goods and people gave Einar the sense that Suðuroy was a place rich with possibilities – he describes feeling like having lived at the developmental centre of the Faroe Islands, due to rapid technological, economic and societal developments. This leads us to suggest that because the world was coming to him, he did not see the need to be geographically mobile. His relative geographical immobility consolidated in the presence of human and non-human mobilities. We therefore want to emphasize that the expansion of the imaginative horizon, through entangled mobilities, does not necessarily lead people to develop an imagination of moving away – it can generate processes of staying as well and still facilitate symbolic mobility.

### Imagining a World Beyond the Island

Einar’s school days came to an abrupt halt as the Second World War reached the Faroe Islands and German air raids began. The local school building was too close to one of the Germans’ main targets – a radio station. Being a young and strong man, as Einar puts it, he quickly found work at the harbour, helping to unload ships’ cargo. Regular fishing halted, but the Faroese fleet started to transport fish from Iceland to Britain, which became a lucrative business. Besides, as Einar jokingly adds, youngsters were faster to run up the mountain when the air alarm sounded.

During this period, the majority of Faroese were practically stuck, yet Einar describes the wartime period with excitement. He recalls working, saving money, and marvelling at the wartime machinery brought across by the British occupational forces. When the war ended, he did not want to return to school and, as many others at the time, Einar stood at a bifurcation point, where prolonged stuckness and decades of contact triggered a longing to go abroad:

After having been […] stuck for five years, people want out. The younger people want out, and then what do you do? Just out and see the world? There wasn’t enough money for that. We should work or study […] There was a lot of Faroese who emigrated, mostly to Denmark, to develop themselves one way or the other.

The post-war years transformed the imaginary horizon in various ways. The proportion of Faroese living on the island of Suðuroy compared to the rest of the nation had begun to decline from the 1920s due to a population boom, though effectively, the islands population first started to drop in the late 1940s to early 1950s.

After the war, people shared a feeling of having been stuck and were hit by an economic crisis in the early 1950s (Guttesen, 1996), leading to a steep increase in emigration. Emigration was, in parts, triggered by the mobility of people and goods observed over the years:

We had a lot of contact with foreigners and naturally got an understanding for what happens abroad. The longing to go abroad have been somewhat bigger on Suðuroy than on the other islands. People think this is part of the explanation [that there was more circulation].

Einar emphasizes that because Suðuroy for many years was a site of high mobility, the islanders had gained new resources that could be used to imagine living elsewhere. The destinations were mostly Tórshavn or Denmark, either to find work or for education – just roaming around was not an option Einar adds. Wealth accumulated during the wartime years and improved infrastructures made it increasingly possible. Spending time abroad almost became a rite of passages for some of those men who did not ship out, and socio-culturally came to represent a way of “developing”, of widening people’s experiences. We suggest that decades of encounters with the mobility of goods, technologies and people contributed to the expansion of the imaginative horizon, to the point where circular mobility entered the realm of possibility and was linked to social mobility (Cohen, 2004).

However, in spite of that, Einar never re-settled outside the island for longer periods of time – even if he wanted to at times. One of the first bifurcation point experienced by Einar after the war pertains to whether or not he should become an angler, arguably one of the pathways with the strongest normative pull for those who did not emigrate:

No, I was not particularly interested but most boys wanted to fish with foreign ships […] it hasn’t been in my blood. If I go back to my youth, I was very seasick. My two brothers had no clue as to what seasickness was. I think that was a small reason that I was not that interested in ships and fishery in that manner. I thought fishery and ships was exiting the whole time, no doubt about that.

For the men living on the island, becoming an angler was “very natural”, Einar adds. While the industry, and all it brought to the island, fascinated him, Einar invokes a propensity for seasickness (“hasn’t been in my blood”) as preventing him from pursuing that path. We may stop to examine this use of a biological justification to support his choice and what it reveals. As said, it seems, in Einar’s environment, fishing was perceived as a “proper” line of work and becoming one was “very natural”. We dare say that the pull towards the ocean was strong back then. Hence, faced with these social expectations – to work as an angler or to move away – Einar appears to have used, consciously or not, an irrefutable argument. After all, he is not opposing the normative expectations by choice, rather his genetic mark-up that does not allow him to work on the ocean, and it is futile to argue against biology.

Due to the diversification of the economy following the Faroese industrialisation (Gaini, 2013; Guttesen, 1996; Joensen, 1987; Wylie, 1987), along with the establishment of more opportunities for schooling (Volckmar, 2019), there were also other, less traversed possibilities. A few years later came a second bifurcation point in Einar’s life. He started to imagine emigrating to Denmark to study. Einar went as far as to arrange an apprenticeship at a blacksmith, through letters, however when his mother caught wind of the plans, she became inconsolable:

[…] my mother, she cried so bitterly for 14 days […] if I left, she would also leave. Not with me, then she did not want to live anymore. But what could we young people do? We could not just walk around doing nothing.

Einar eventually cancelled his plans and chose to stay on the island, despite the fact that, as he said, he could just have left and blamed it on being young, but “that was not me”, Einar adds. He reckons that this decision disappointed his father a bit, who had hoped for Einar to move abroad to “develop”. In fact, none of the three brothers moved away from the island. So, when Einar started to imagine becoming a blacksmith and began to plan transnational mobility, his social and affective relations delegitimises that imagination (Zittoun & Gillespie, 2016) and consolidates his staying. We know from other research that social and affective ties can both mobilise and immobilise people (e.g. Cole & Groes, 2017; Coulter et al., 2016; Mata-Codesal, 2015; Schapendonk, 2020; Stockdale et al., 2018), as well as enable, guide, or restrict what people imagine and if it can be actualised. This also echoes Elder et al. (2003) argument that lives are inevitably linked together and interdependent, which entails that social relationships are crucial in shaping transitions and, more generally, how the life course unfolds. In Einar’s case, his father, along with the more general normative pull equating mobility with “development” was overruled, in parts, by his close relation to his mother, his two brothers (Einar often speak about the three of them as a little group), and Tvøroyri. As Stockdale et al. (2018), Hjälm (2014), and others have shown, staying is intimately linked to family relations, and the fact that none of them moved also influenced Einar’s decision to remain on the island despite imagining moving to Denmark. He faced the difficult task of negotiating between two somewhat contradictory forces, but, ultimately, because he never felt stuck on Suðuroy and always enjoyed his close social and affective relations, he ended finding a middle-way – one in which he could be around his mom and brothers and “develop” through other means than his own geographical mobility.^1^

Einar was able to get an apprenticeship at a local clerk’s office, with help from his father’s connections. Here he immersed himself in local history, reading, among others, detailed accounts of the distribution of whale meat. Meanwhile, he also took evening classes in commerce and practiced his English skills through megaphone courses. The money that Einar had saved during the war and which had originally been earmarked for going to Denmark, he used to buy a plot of land where he could construct a house. He reckons this was quite the achievement at the time, because, just a generation ago, this would have been an impossible imagination for many. In other words, after renouncing to geographical mobility in favour of staying, Einar further consolidated his relative immobility through buying land in the village and committed to his family and social network, while seemingly also climbed the social ladder.

In this period, we suggest that the imaginative horizon of Suðuroy expanded. Circulation of capital, people and goods during the 1930s and 1940s, wealth accumulation, feelings of being trapped, and more created pathways leading to Denmark and sometimes beyond. On the other, the fishing industry and another way of life still led to certain pathways appearing more “natural’ than others. Young men, like Einar, faced social expectations to become an angler or to move away to “develop”, but he stayed and did not become an angler. At the first bifurcation point, Einar uses innate, biological conditions to resist “sailing out” and remains relatively geographically immobile. The second bifurcation brings him to align his imagination with the imaginative horizon – to go abroad to study – yet here the risk of cutting close affective and social ties renders that imagination impossible and Einar choses to stay in the village. His decision to stay is linked to the (still) lives of other people, particular his mother and two brothers. We thus saw Einar deploying his imagination partly in resistance to the imaginative horizon; yet were sociocultural norms can be resisted, perhaps social and affective ties less so. Also, we observed how he could use various social, cultural and financial resources to transform geographical immobility into imaginary and social mobility – and as we will see, further symbolic mobility.

### Being on the Move while Remaining Still

Wartime isolation from Denmark gave an extra momentum to a wave of nationalism that had been building since the late 19-century (Sølvará, 2016), which eventually culminated in a home-rule system a few years after the war. It transferred some aspects of governance to a local Faroese Government in 1948 while others stayed in Copenhagen.

For Einar, this geo-political change opened a new field of possibilities locally: He became one of the nation’s first five customs officers starting in 1949. This placed him at the very juncture of incoming ships, anglers and goods. Einar chose a profession connected to the fishing industry, which the imaginative horizon was, to a degree, centralised around, allowing him to maintain a life-long interest in ships and everything they brought with them without becoming an angler. Here Einar found many resources to nourish his imagination and, hence, symbolic mobility. Though the industry was struggling to modernise in the 1950s, there was amble mobility in and through Suðuroy:

There was a need for customs officers almost 24/7 […] because there were foreign shops […] and many people on board. So, there was a tremendous development on that front, and I learned a lot on one or the other hand.

Einar’s position at the juncture of mobility provided him the opportunity to learn from the countless encounters with anglers from distant places (Glăveanu, 2020a), while staying relatively geographically immobile himself. For instance, the ferry Torshavn took 4 hours each way, under ideal conditions, and travelling to Denmark took 50 h across the Atlantic Ocean. Einar first visited Denmark in 1951, to visit his wife who was there for some training, and instead of stirring up old imaginations of moving away from Suðuroy to become a blacksmith, it supported his decision to stay. All the encounters with foreign anglers and increased ability to travel enabled him to stay. He was aware of the tension between the pressure to move away in order to develop:

[…] I do not why we [him and his brothers] are so stupid not develop our foreign ways, but that is our little problem, but we are glad for that problem.

Einar is not sad about their supposedly lack of “development” – of not having moved away to broaden their perspective – in fact, he is happy to have stayed on the island with his brothers. Once again, part of what allows Einar to resist going abroad, even in times when the sociocultural meaning of mobility was linked to ideas of personal development (Cresswell, 2010), is his social and affective ties. He prioritises staying and family relations over the normative pull to become mobile. Einar’s staying correspond to what the studies, mentioned earlier, demonstrate in regard to attachment to local community and socio-economic and affective circuits, local imaginaries that infuse relative geographical mobility with meaning, and many more to produce a desired immobility (Carling, 2014; Carling & Collins, 2018; De Haas, 2014). For Einar, relative geographical immobility almost becomes a virtue in itself. However, as we also showed, the expanding imaginative horizon might still have turned his gaze and imagination outwards, leading him to develop an immense curiosity for the world beyond. In particular, Einar expresses interest in understanding why the Faroese, according to him, appear technologically and socially lagging behind – a question that in itself is a testimony to his symbolic mobility, as he imagines how things might have been otherwise.

We propose that this entanglement of the mobility anglers, technologies, and ideas and the relative geographical immobility of his family are what facilitated Einar’s staying. Here we also suggest that his language skills played a central part:

I was so happy that I had developed my English quite well […] and I was happy because I really liked being around people – foreign people […] And there was so much ship traffic and I was really happy about it.

Being able to communicate is what enables Einar to have encounters and conversations with mobile others, and, hence, increase his symbolic mobility. Einar told Oliver that he listened to tape recordings to improve his English, which, in turn, was further developed communicating with people from incoming ships, as part of his work as custom officer. English gave him access to an expanding new symbolic universe that the anglers brought with them, widening the resources Einar has for imagining, through hearing about their journeys, distant home regions, and ways of conducting business. Another interesting and related example is how Einar prides himself being able to tell the many Scottish dialects apart and thus starts to build a comprehensive imagination of Scotland abstracted from individual anglers:

And I dare say that I bragged a bit about from the Scottish anglers who arrived, I could point out after three years together with them […] when a new man came on board a ship, fishing ship, he was that and that little place in Scotland, because of the dialects.

Talking to incoming anglers appears to have been an important way of exploring the world for Einar. Without language skills, his imagination of, say Scotland and its anglers, would likely be impoverished and less differentiated. However, after three years of working as a custom officer, Einar progressive built a differentiated imagination of Scotland to the point where he is able to tell individual anglers apart and match them with specific villages, simply of their dialects. On a more general level, encounters with technologies and people spark Einar’s imagination, and enabled his exploration of the world from afar. For instance, talking with foreign anglers and inspecting the latest equipment enabled Einar to be symbolically mobile, to explore what is absent and “develop”:

Because there were foreign ships and foreign docks, and so many people on board […] so I learned a lot one way or the other.

Through these encounters, Einar could imagine distant places as well as alternatives to his present and local life – all without having to be geographically mobile himself – for instance, learning about the construction of salt silos in the Mediterranean:

With the ships from the Mediterranean, it was incredibly interesting to hear how they develop salt mines and how they built salt silos different places to sell.

We argue that arrivals of ships and anglers also became aspects engendering Einar’s staying on the island, because it allowed him to travel imaginatively – through the stories of foreign anglers, merchants, and the like – while not necessarily visiting these places. In other words, these mobilities mediated the relative immobility of Einar’s family and his curiosity for the world. Furthermore, we observe that through language acquisition, Einar obtained access to a different symbolic universe that he transformed into resources for imagining, moving gradually from the concrete to the abstract. Following Vygotsky (2004), the numerous encounters and conversations seemed to have enriched Einar’s experience and thus his imagination. This exemplifies how Einar built complex imaginations of the elsewhere and is mobile through the imagination, yet without necessarily leading him to more mobile. It also demonstrates that his staying is deeply entangled with other forms of mobility and immobility, which both expand his imagination and at the same time, supports his relative geographical immobility.

## Openings: Imagination and Staying

In this paper we proposed to contribute to the sociocultural psychology study of mobility by focusing on story of Einar. Articulating sociogenetic transformation of the imaginary horizon with the ontogenetic course of life, we could identify some of the processes by which Einar’s imagination developed in and through (im)mobilities, in changing social circumstances. Einar life took place in times characterised by an expansive imaginative horizon, high levels of emigration, and imperatives to journey abroad to “develop”. However, we showed how Einar’s imagination of what lies beyond the island increased and became progressively differentiated though his encounters with incoming technologies, ideas, and anglers, which could be used as resources in his symbolic mobility. Einar seems to have felt that the world was coming to him and that he could explore it from afar. Thus, we believe, Einar’s story demonstrates that, first, imagining life elsewhere does not necessarily trigger geographical mobility, but it may also equally engender processes of staying. Second, it shows that staying is an active process unfolding throughout people’s life trajectories, and which can neither be separated from its entanglement in a web of human as well as non-human mobilities, nor from individual socio-economic positions, experiences, social and affective ties, and imagined futures. Social and affective relations appeared to have counteracted the imperatives to be mobile and delegitimised Einar’s imaginations of becoming a blacksmith, further underline his decision to stay. Studying the relation between mobility, immobility and imagination along people’s trajectories highlight the many negotiations and ambivalences involved in the process.

We suggest that differentiating between symbolic and geographical mobility and immobility contributes to mobility studies, as it offers an analytical tool to unpack the imaginative processes that both triggers, and are triggered by, movement and stasis of the self as well as others, technologies, and ideas. Further, we argue that exploring this entanglement requires a contextual, perspectival and dynamic approach. Here we propose that the notion of imaginative horizons can capture different forces impinging on people’s imagination and (im)mobility throughout time, while still recognising that people are uniquely located in these situations, and draw upon available socioeconomic and symbolic resources, to feed their symbolic mobility. In short, we demonstrate the link between processes of staying and other, entangled, mobilities at the intersection of imaginative horizons and unique individual trajectories of imagining.

Generalising from this case study also has implications for sociocultural psychology (Flyvbjerg, 2011; Zittoun, 2017). We invite sociocultural psychologists to expand their understanding of human development by paying more attention to its entanglement with human as well as non-human (im)mobilities. Mobility studies give a depth to the analysis of the historico-cultural context and its evolution, by inviting to examine geographical movements which expand and constrain the field of what is possible or imaginable (Glăveanu, 2020a; Hawlina et al., 2020; Märtsin, 2019). They also invite us to examine the role of moving or staying in people’s life courses. In addition, our analysis opens new avenues in the study of human development across the life course by examining the expansion and centralisation of imaginative horizons. In addition, it opens questions about how imagination itself develops through movements of the self and of others, which can be used as resources to build progressively more differentiated semiotic systems, and to elaborate complex distant imaginations (Gillespie & Zittoun, 2013), which can both incentivise geographical and symbolic (im)mobility as well as re-orient life courses.

Increasingly differentiated imaginations of distant places or alternative presents are more than the lure of a better future promised by and incentivising mobility – it also engenders staying. There is a need to ask what imagination does, in what situations, and for whom, if we want to understand people’s (im)mobility across times and spaces. We need to account for the different entanglements of mobilities, imaginative horizons, social and affective relations, and unique life trajectories when studying different capacities to imagine and to capture that the promise of being mobile for one person might mean staying for another.

## Footnotes

1 We acknowledge that the interdependence of Einar’s mobility and imagination are inseparable from that of his family relation, however, due to COVID-19, we were unfortunately not able of exploring this dimension more in-depth.

## Declarations

No declarations.

## References

[CR1] Adey P (2006). If mobility is everything then it is nothing: Towards a relational politics of (im)mobilities. Mobilities.

[CR2] Adey, P. (2017). *Mobility* (2nd ed.). Routledge. 10.1017/CBO9781107415324.004.

[CR3] Anderson, B. (2019). New directions in migration studies: towards methodological de-nationalism. *Comparative Migration Studies*, *7*(36). 10.1186/s40878-019-0140-8.

[CR4] Appadurai A (1996). Modernity at Large: Cultural Dimensions of Globalization.

[CR5] Baas, M. (2010). *Imagined mobility: Migration and transnationalism among Indian students in Australia*. 10.7135/UPO9781843313410.

[CR6] Barcus HR, Brunn SD (2009). Towards a typology of mobility and place attachment in rural America. Journal of Appalachian Studies.

[CR7] Benson M (2012). How culturally significant imaginings are translated into lifestyle migration. Journal of Ethnic and Migration Studies.

[CR8] Brandt J (1983). Kapitalismens Udvikling På Færøerne. Geografisk Orientering.

[CR9] Brú, H. (2011). *The old man and his sons*. Telegram Books.

[CR10] Bruner J (1990). Acts of meaning.

[CR11] Cangià F (2020). (Im)Mobile imagination. On trailing, feeling stuck and imagining work on-the-move. Culture and Psychology.

[CR12] Cangià F, Zittoun T (2020). Exploring the interplay between (im)mobility and imagination. Culture and Psychology.

[CR13] Carling J (2002). Migration in the age of involuntary immobility: Theoretical re¯ections and Cape Verdean experiences. Journal of Ethnic and Migration Studies.

[CR14] Carling, J. (2014). The role of aspirations in migration. *Paper Presented at Determinants of International Migration, International Migration Institute, University of Oxford*, *September*, 23–25. 10.1016/j.soscij.2012.07.006.

[CR15] Carling J, Collins F (2018). Aspiration, desire and drivers of migration. Journal of Ethnic and Migration Studies.

[CR16] Carling J, Schewel K (2018). Revisiting aspiration and ability in international migration. Journal of Ethnic and Migration Studies.

[CR17] Cohen JH (2004). The Culture of Migration in Southern Mexico.

[CR18] Cole J, Groes C, Cole J, Groes C (2017). Affective Circuits: African Migrations to Europe and the Pursuit of Social Regeneration. Introduction: Affective Circuits and Social Regeneration in African Migration.

[CR19] Coulter R, van Ham M, Findlay AM (2016). Re-thinking residential mobility: Linking lives through time and space. Progress in Human Geography.

[CR20] Crapanzano, V. (2004). *Imaginative Horizons: An Essay in Literary-Philosophical Anthropology*. The University of Chicago Press.

[CR21] Cresswell T (2001). The production of mobilities. New Formations.

[CR22] Cresswell T (2006). On the move: Mobility in the modern western world. Routledge.

[CR23] Cresswell T (2010). Towards a politics of mobility. Environment and Planning d: Society and Space.

[CR24] Cresswell T (2012). Mobilities II: Still. Progress in Human Geography.

[CR25] Dahinden J (2016). A plea for the ‘de-migranticization’ of research on migration and integration. Ethnic and Racial Studies.

[CR26] De Haas, H. (2014). Migration Theory Quo Vadis? *The IMI Working Papers Series*, *November*, 1–39.

[CR27] Easthope H (2009). Fixed identities in a mobile world? The relationship between mobility, place, and identity. Identities.

[CR28] Elder GH, Giele JZ (2009). The Craft of Life Course Research.

[CR29] Elder, G. H., Johnson, M. K., & Crosnoe, R. (2003). The emergence and development of life course theory. In J. T. Mortimer & M. J. Shanahan (Eds.), Handbook of the life course (pp. 3–19.). Kluwer Academic/Plenum.

[CR30] Faist T (2013). The mobility turn: A new paradigm for the social sciences?. Ethnic and Racial Studies.

[CR31] Ferguson J, Gupta A (1992). Beyond " Culture ": space, identity, and the politics of. Cultural Anthropology.

[CR32] Fischer PA, Malmberg G (2001). Settled people don’t move: On life course and (Im-) mobility in Sweden. International Journal of Population Geography.

[CR33] Flyvbjerg, B. (2011). Case study. In N. K. Denzin, & Y. S. Lincoln (Eds.), *The Sage Handbook of Qualitative Research* (4th ed., pp. 301–316). Sage.

[CR34] Foucault M, Richardson J, Leiter B (1978). Nietzsche, Genealogy, History. Nietzsche.

[CR35] Gaffin D (1996). In Place: Spatial and Social Order in a Faeroe Islands Community.

[CR36] Gaini F (2013). Lessons of Islands: Place and identity in the Faroe Islands. Faroe University Press.

[CR37] Gillespie A (2006). Becoming Other: From Social Interaction to Self-reflection.

[CR38] Gillespie A, Zittoun T (2013). Meaning making in motion: Bodies and minds moving through institutional and semiotic structures. Culture and Psychology.

[CR39] Gillespie A, Zittoun T, Wagoner B, Chaudhary N, Hviid P (2015). Social and psychological movement: Weaving individual experience into society. Integrating experiences: Body and mind moving between contexts.

[CR40] Glăveanu VP (2020). Mobilities and Human Possibility.

[CR41] Glăveanu VP (2020). The Possible: A Sociocultural Theory.

[CR42] Glick Schiller N, Salazar NB (2013). Regimes of mobility across the globe. Journal of Ethnic and Migration Studies.

[CR43] Griffiths MBE (2014). Out of time: The temporal uncertainties of refused asylum seekers and immigration detainees. Journal of Ethnic and Migration Studies.

[CR44] Guttesen, R. (1996). *The Faroe Islands Topographic Atlas* (K. N. Jacobsen, Ed.). the Royal Danish Society of Geography.

[CR45] Haas Hde, Fransen S (2018). Social transformation and migration: An empirical inquiry. IMI Working Paper Series.

[CR46] Hage, G. (2009). Waiting out the crisis: On stuckedness and governmentality. *Waiting*, 1–9.

[CR47] Halfacree KH, Boyle PJ (1993). The challenge facing migration research: The case for a biographical approach. Progress in Human Geography.

[CR48] Hannam K, Sheller M, Urry J (2006). Editorial: Mobilities, immobilities and moorings. Mobilities.

[CR49] Hawlina, H. (2019, August 19). *Collective imagination and sociogenesis: Envisioning alternatives under societal constraints* [Oral presentation]. 18th Conference of the International Society for Theoretical Psychology, Copenhagen.

[CR50] Hawlina H, Pedersen OC, Zittoun T (2020). Imagination and social movements. Current Opinion in Psychology.

[CR51] Heil, T., Priori, A., Bruno, R., & Schwarz, I. (2017). Mobilities – Migratory Experiences Ethnographically Connected: An Introduction. *New Diversities 19*, *19*(3).

[CR52] Hjälm A (2014). The ‘Stayers’: Dynamics of lifelong sedentary behaviour in an urban context. Population, Space and Place.

[CR53] Holm, D., & Mortensen, B. (2002). Economic life on the periphery: A comparative study of economic development in two towns on Suduroy. In R. Apostle, D. Holm, G. Hovgaard, Ó. Waag Høgnesen, & B. Mortensen (Eds.), *The Restructuration of the Faroese Economy: The Signifi cance of the Inner Periphery* (pp. 25–74). Samfundslitteratur Press.

[CR54] Hviid, P., & Villadsen, J. W. (2015). Ruptures and repairs in the course of living: Challenges to developmental psychology. In Joerchel. A. C., & G. Benetka (Eds.), *Biographical ruptures and their repair. Cultural transitions in development* (pp. 57–81). Information Age Publishing.

[CR55] Ingold, T. (2008). Bindings against boundaries: Entanglements of life in an open world. *Environment and Planning A*, *40*(8). 10.1068/a40156

[CR56] Joensen, J. P. (1987). *Fra bonde til fisker: Studier i overgangen fra bondesamfund til fiskersamfund på Færøerne*. Føroya Fornminnissavn.

[CR57] Jónsson, G. (2011). Non-migrant, sedentary, immobile , or ‘ left behind ’? Reflections on the absence of migration. *The IMI Working Papers Series*, *April*, 1–17.

[CR58] Kalir B (2013). Moving subjects, stagnant paradigms: Can the ‘Mobilities Paradigm’ Transcend Methodological Nationalism?. Journal of Ethnic and Migration Studies.

[CR59] Khosravi S (2010). ‘Illegal’ Traveller—An autoethnography of borders. Palgrave Macmillan.

[CR60] Kleist N (2020). Follow the computers: Entangled mobilities of people and things in transnational recycling. Ethnography.

[CR61] Kleist N, Jansen S (2016). Introduction: Hope over time—crisis, immobility and future-making. History and Anthropology.

[CR62] Kohler-Riessman, C. (2000). Analysis of personal narratives. *Qualitative Research in Social Work*, 168–191.

[CR63] Looker, D. E., & Naylor, T. D. (2009). ‘At Risk’ of Being Rural? The Experience of Rural Youth in a Risk Society. *Journal of Rural and Community Development*, 39–64.

[CR64] Malkki L (1992). National geographic: The rooting of peoples and the territorialization of National Identity Among Scholars and Refugees. Cultural Anthropology.

[CR65] Mar P (2005). Unsettling potentialities: Topographies of hope in transnational migration. Journal of Intercultural Studies.

[CR66] Marková I, Zittoun T, Glaveanu V (2017). From Imagination to Well-Controlled Images: Challenge to the Dialogical Mind. Handbook of Imagination and Culture.

[CR67] Marková I, Zadeh S, Zittoun T (2020). Introduction to the special issue on generalisation from dialogical single case studies. Culture and Psychology.

[CR68] Märtsin M (2019). Identity Development in the Lifecourse: A Semiotic Cultural Approach to Transitions in Early Adulthood.

[CR69] Massey D (2005). For Space.

[CR70] Mata-Codesal D (2015). Ways of staying put in Ecuador: social and embodied experiences of mobility-immobility interactions. Journal of Ethnic and Migration Studies.

[CR71] Mata-Codesal D (2018). Is it simpler to leave or to stay put? Desired immobility in a Mexican village. Population, Space and Place.

[CR72] Mulder CH, Malmberg G (2014). Local ties and family migration. Environment and Planning A.

[CR73] Numminen, L. (2010). *the Interplays of Histories , Economies and Cultures in Human Adaptation and Settlement Patterns: The Cases of the Faroe Islands and Greenland* (Issue Yliopistonkatu 3).

[CR74] Pine F (2014). Migration as hope: Space, time, and imagining the future. Current Anthropology.

[CR75] Rérat P (2014). The selective migration of young graduates: Which of them return to their rural home region and which do not?. Journal of Rural Studies.

[CR76] Rosa, A., & Valsiner, J. (2018). *Socio-Cultural Psychology on the Move Semiotic Methodology in the Making*. 10.1017/CBO9780511611162.038.

[CR77] Salazar, N. B. (2010). *Envisioning Eden: Mobilizing imaginaries in tourism and beyond*. Berghahn Books.

[CR78] Salazar NB (2011). The power of imagination in transnational mobilities. Identities.

[CR79] Salazar, N. B. (2014). Migrating Imaginaries of a Better Life … Until Paradise Finds You. *Understanding Lifestyle Migration*, 119–138. 10.1057/9781137328670_6.

[CR80] Salazar NB (2020). On imagination and imaginaries, mobility and immobility: Seeing the forest for the trees. Culture and Psychology.

[CR81] Sato, T., & Valsiner, J. (2010). *Time in Life and Life in Time: Between Experiencing adn Accounting*. *2010*, 79–92.

[CR82] Sato, T., & Yasuda, Y. (2013). *From Describing to Reconstructing Life Trajectories :*

[CR83] Schapendonk, J. (2020). *Finding ways through Eurospace: West African Movers Re-viewing Europe from the Inside*. Berghahn Books.

[CR84] Schapendonk, J., van Liempt, I., Schwarz, I., & Steel, G. (2018). Re-routing migration geographies: Migrants, trajectories and mobility regimes. *Geoforum*, *June 2017*, 0–1. 10.1016/j.geoforum.2018.06.007.

[CR85] Schewel, K. (2019). Understanding Immobility: Moving Beyond the Mobility Bias in Migration Studies. *International Migration Review*, 1–28. 10.1177/0197918319831952.

[CR86] Sheller M, Urry J (2006). The new mobilities paradigm. Environment and Planning A.

[CR87] Söderström, O., Randeria, S., Ruedin, D., D’Amato, G., & Panese, F. (2013). Of Mobilities and Moorings: Critical Perspectives. *Critical Mobilities*, *March*, V–XXXIV.

[CR88] Sølvará, H. A. (2016). The Rise of Faroese Separatism: Danish-Faroese relations from 1906-1925 and the radicalization of the national- and home rule question. (1 ed.) (Supplementum; No. 66). Fróðskapur.

[CR89] Sølvará HA (2020). Færøerne efter freden.

[CR90] Stockdale, A., & Haartsen, T. (2018). Editorial introduction: Putting rural stayers in the spotlight. *Population, Space and Place*, *24*(4). 10.1002/psp.2124.

[CR91] Stockdale A, Theunissen N, Haartsen T (2018). Staying in a state of flux: A life course perspective on the diverse staying processes of rural young adults. Population, Space and Place.

[CR92] Urry J (2007). Mobilities.

[CR93] Valsiner, J. (1998). *The guided mind: A sociogenetic approach to personality*. Harvard University Press.

[CR94] Valsiner J (2007). Culture in Minds and Societies: Foundations of Cultural Psychology.

[CR95] Vigh H (2009). Wayward migration: On imagined futures and technological voids. Ethnos.

[CR96] Volckmar N (2019). The faroese path to a comprehensive education system. Nordic Journal of Educational History.

[CR97] Vygotsky L (2004). Imagination and creativity in childhood. Journal of Russian and East European Psychology.

[CR98] West JF (1972). Faroe: The emergence of a nation.

[CR99] Wimmer A, Glick Schiller N (2002). Methodological nationalism and beyond: Nation-state building, migration and the social sciences. Global Networks.

[CR100] Womersley, G. (2020). (Un)imagination and (im)mobility: Exploring the past and constructing possible futures among refugee victims of torture in Greece. *Culture and Psychology*, *im*, 1–19. 10.1177/1354067X19899066.

[CR101] Wylie J (1987). The Faroe Islands: Interpretations of history.

[CR102] Wyss A (2019). Stuck in mobility? Interrupted journeys of migrants with precarious legal status in Europe. Journal of Immigrant and Refugee Studies.

[CR103] Ye J (2018). Stayers in China’s “hollowed-out” villages: A counter narrative on massive rural–urban migration. Population, Space and Place.

[CR104] Zittoun T (2017). Modalities of generalization through single case studies. Integrative Psychological and Behavioral Science.

[CR105] Zittoun T (2020). Imagination in people and societies on the move: A sociocultural psychology perspective. Culture & Psychology.

[CR106] Zittoun, T., & Gillespie, A. (2016). Imagination in human and cultural development. In *Imagination in Human and Cultural Development*. Routledge. 10.4324/9780203073360.

[CR107] Zittoun T, Gillespie A, de Saint-Laurent C, Obradovic S, Carriere KR (2018). Imagining the collective future: A sociocultural perspective. Imagining Collective Futures.

[CR108] Zittoun, T., Hawlina, H., & Gillespie, A. (2020). Imagination. In *The Palgrave Encyclopedia of the Possible* (pp. 1–8). Springer International Publishing. 10.1007/978-3-319-98390-5_68-1

[CR109] Zittoun T, Valsiner J, Vedeler D, Salgado J, Gonçalves M, Ferring D (2013). Human development in the lifecourse: Melodies of living.

